# Editorial Correspondence

**Published:** 1877-10

**Authors:** 


### EDITORIAL CORRESPONDENCE.

No. 2214 Chestnut St., Philadelphia, Pa.,
October 2, 1877.

*Mr. Editor:*

A South Carolinian by birth and education, I send to yours as the representative Journal of the Southern States, and submit to your consideration the following practical hints on the therapeutics of phthisis, for publication. Although perhaps not presenting any new fact, I think they will serve as a good reminder of the most trustworthy therapeutical agents we have in phthisis. I have refrained from a strong indorsement of Dr. Polk's glycerite of kephaline, but I am very much pleased with the results I have obtained from it, and hope to see it tested by the profession. * * *

Phthisis is yet considered an incurable malady. No remedy has yet been found that will certainly stay its ravages, and the baffled physician is inclined to think all treatment useless. I do not think, however, that an examination of the subject justifies such a melancholy view. Although we cannot always cure consumption, we can already do much towars delaying its progress, and by the timely adoption of hygienic regulations, do still more towards preventing the generation or devlopment of the tubercular diathesis. As phthisis is a disease of general vital deterioration, the remedy must be found in means calculated to exalt the organic functions of the individual, and obviate all depressing agencies, whether they relate to the body or the brain. Outdoor excercise is a hackneyed injunction, and yet one that is too much disregarded. In the therapeutics of tubercular phthisis, there is nothing equal to it; we, therefore, assign it the first place. Well ventilated sleeping apartments is another well-recognized requisite, written on in every treatise on tuberculosis, and yet not sufficiently enforced. Fresh air by day and night, and outdoor exercise are the great remedies both in the prevention and cure of tubercular phthisis; they must not be overlooked.

Rich nutritious food is another important item, and it should embody the saccharine principles, the phosphates, and the hypophosphites, demanded in brain organic and physical processes. A fact we owe to Dr. Polk, I believe, is that one of the earliest lesions of phthisis is the substitution of butyric for lactic acid, and, as Professor Samuel Jackson pointed out, is an indispensable requisite of secondary or tissue digestion. About seven years ago Dr. Polk demonstrated by experiments on dogs (see "Tuberculosis," New Orleans *Medical and Surgical Journal* for September, 1877,) that lactic acid endows ordinary phosphate of lime with sufficient organismal power to supply the phosphates to the system of an animal. As butyric acid is but a consequence of indigestion, we should try to correct this. I have had good results from nitromuriatic acid and compound tincture of cinchonia. These seem to do better than anything I have tried in obviating the formation of butyric acid. In one case of incipient phthisis they, with the dietic and hygienic management alluded to, seemed to secure a perfect restoration to health. Sulphate of quinine, with nitric acid, is to be substituted when the nitromuriatic acid causes pain in the stomach. If the patient be somewhat anæmic, and no inflammation present, or strong tendency to hemorrhage, I often use this combination with excellent results:

℞.—Acid. nitrici dil.,

Tinct. ferri chl., aa., . . . . . ℥ ss

Sulph. quinia, . . . . . . . ʒ j M.

Sig.—Twenty drops thrice daily.

I cannot agree with Dr. A. P. Dutcher, that we can well dispense with cod liver oil. I am sure I have witnessed very satisfactory results from its employment. I usually combine it with extract of malt, and add syrup of the lacto-phosphate of calcicum to the combination. Upon this treatment I have seen several phthisical patients gain strength and flesh. I regard it as an excellent association of remedial agents. I prefer the white Norwegian oil, and when I cannot get this, resort to the bottled oil of Marvin.

I have used the compound syrup of the hypophosphites with benefit, in several cases, and regard them worthy of trial. I believe, in conjunction with cod liver oil, they will cure a considerable number of cases in the earlier stages, although utterly useless in the more advanced stage. During the past six months, I have used the brain cerebrates, or hypophosphites, in several cases, and I am well pleased with the results. They seem to be superior to the laboratory-made hypophosphites, and I believe they are worthy of general investigation. As the mode of isolation, and the formula, were published in the October number of the *Druggist Circular*, the objections as to "Glycerite of Kephaline" being a proprietary or secret nostrum cannot be longer entertained. I believe it can be obtained in Louisville, St. Louis, New York, and most of the large cities.

